# External Auditory Canal Cholesteatoma after Radiation Therapy for Nasopharyngeal Cancer: Case Series and Systematic Review

**DOI:** 10.3390/jcm12051977

**Published:** 2023-03-02

**Authors:** Fulvio Mammarella, Antonella Loperfido, Michele Cianciulli, Bruno Fionda, Alessandro Stasolla, Gianluca Bellocchi

**Affiliations:** 1 Otolaryngology Unit, San Camillo Forlanini Hospital, 00152 Rome, Italy; 2 Department of Radiation Oncology, San Camillo Forlanini Hospital, 00152 Rome, Italy; 3 U.O.C. Radioterapia Oncologica, Fondazione Policlinico Universitario Agostino Gemelli IRCCS, 00168 Rome, Italy; 4 Neuroradiology Unit, San Camillo Forlanini Hospital, 00152 Rome, Italy

**Keywords:** external auditory canal cholesteatoma, EACC, radiotherapy, radiation therapy, nasopharyngeal cancer

## Abstract

The authors performed a systematic review, in accordance with the PRISMA guidelines, across multiple databases, including all original studies published until November 2022, focusing on External auditory canal cholesteatoma (EACC) after radiation therapy (RT) for nasopharyngeal cancer (NC). Inclusion criteria were original articles reporting on secondary EACC after RT for NC. Articles were critically appraised to assess level of evidence using the Oxford Center for Evidence-Based Medicine criteria. Overall, 138 papers were identified and after duplicate removal (34 papers) and excluding papers not in English, 93 papers were assessed for eligibility; finally, only five papers were included and summarized with the three cases coming from our institution. These mainly involved the anterior and the inferior part of the EAC. The mean time of diagnosis after RT was the largest series of 6.5 years (with a range from 0.5 to 15.4 years). Patients undergoing RT for NC have 18 times a higher risk of developing EACC compared to the normal population. EACC is probably one of the most underreported side effects, because patients may present variable clinical findings, which could lead to misdiagnosis. Early diagnosis of RT related EACC is advised to enable conservative treatment.

## 1. Introduction

External auditory canal cholesteatoma (EACC) represents a very uncommon disease [1], with a reported incidence of 1 in 1000 new otologic patients [2]; on the other hand, radiation therapy (RT) represents a key therapeutic strategy to treat nasopharyngeal cancer [3].

Available epidemiologic data allow an estimation that patients undergoing RT for nasopharyngeal cancer have a 18 times higher risk of developing EACC compared to a normal population [4].

First described by Toynbee in 1850 [5], EACC is defined in the literature as an accumulation of keratin leading to osteolytic bony erosion [6].

In 1980 this clinical entity was well defined and differentiated from keratosis obturans. Keratosis obturans usually presents as hearing loss and acute pain due to the accumulation of large plugs of desquamated keratin in the auditory canal. EACC has a different mode of presentation leading to otorrhea, with a chronic and dull pain which is consequent to an invasion of squamous tissue into a localized area of periositis in the canal wall [7].

The recent technological advances in RT, with consequent therapeutic gains in terms of prognosis for patients affected by head and neck cancer, come with an increased possibility of RT related long-term side effects: xerostomia, osteoradionecrosis, fibrosis, skin changes and ear structures damage [8].

Even though not very frequent, EACC is probably one of the most underreported side effects in clinical practice [9], because patients may present variable clinical findings, which could lead to misdiagnosis [10].

The diagnostic criteria proposed in the literature for RT-related EACC are clinical and pathological evidence of the accumulation of keratin debris, hyperplasia of the external auditory canal epithelium, and bony erosion [4].

We present three patients affected by EACC and who had a history of nasopharyngeal carcinoma treated with RT alone or combined with chemotherapy. We also present the first systematic review on this topic.

## 2. Materials and Methods

All patients were treated according to the principles of good clinical practice and signed written informed content for publication of their cases. The systematic review portion of this study was performed according to Preferred Reporting Items for Systematic Review and Meta-Analysis guidelines [11]. No published review protocol exists for this topic.

### 2.1. Search Strategy

A systematic review of all articles present in the main medical databases, such as PubMed (NLM NIH), Scopus (Elsevier and Cochrane library (Wiley)), was performed. The time period considered included all the published articles available within the databases from their inception until November 2022. Additionally, a manual search of the relevant literature in otolaryngology and radiotherapy meetings and citation chaining were performed in order not to miss any relevant case.

### 2.2. Selection Criteria

The search strategy included a combination of the following terms: “Radiotherapy” or “Radiation Therapy” and “Cholesteatoma”. The inclusion criteria were original articles specifically reporting on secondary EACC (external auditory canal cholesteatoma) after RT for NPC (nasopharyngeal cancer), including both prospective and retrospective studies. Reviews, letters to the editor, articles not in English, conference papers, and papers with mixed cases other than NPC were excluded. Two independent authors, a radiation oncologist (BF) and an otorhinolaryngologist (AL), screened citations in titles and abstracts in order to identify appropriate papers. Eligible citations were retrieved for full-text review. 

### 2.3. Quality Assessment

Articles were critically appraised to assess level of evidence using the Oxford Center for Evidence-Based Medicine criteria [12].

### 2.4. Data Extraction

The extracted data included: author, publication year, country, number of patients, age, sex, RT dose, RT technique, time from RT to EACC diagnosis, side, and location within the EAC.

### 2.5. Biostatistics

Data from the selected cases were collated and processed using the Data Analysis Tool Pak loaded in Excel to calculate descriptive statistics.

## 3. Results

### 3.1. Patient 1

In May 2022 a 57-year-old male presented with a history of left otorrhea and recurrent ear pain for about ten months. He had already undergone several courses of antibiotic therapy on the recommendation of his general practitioner without benefit. He also reported a history of recurrent bilateral ear infections from a young age and a known bilateral hearing loss, more severe on the left.

The patient also reported a history of nasopharyngeal undifferentiated carcinoma diagnosed when he was 45 and treated with RT with a dose of 66 Gy in 30 fractions.

**Figure 1:**
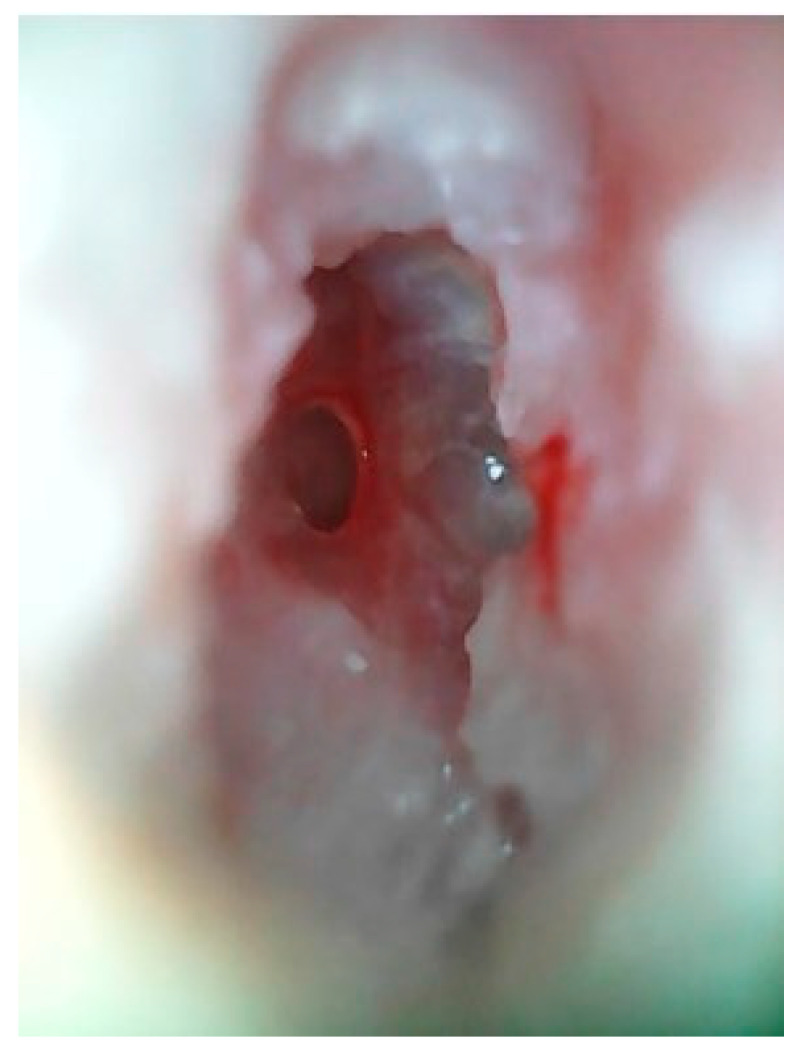
Patient 1, Otomicroscopy: evidence of erosion of the anteroinferior wall of the left EAC and a small anterior tympanic perforation.

Physical examination with otomicroscopy revealed purulent discharge and white skin-like material in the left EAC. The patient underwent ear cleaning under microscopic guidance, finding hyperplasia of the EAC mucosa, an erosion of the anteroinferior wall of the left EAC and a small anterior tympanic perforation, as shown in Figure 1.

The audiometric exam revealed a moderate degree of neurosensory hearing loss on the right (PTA equal to 58 dB HL) and a profound degree on the left (PTA equal to 93 dB HL), with bilateral loss of frequencies above 2000 Hz.

**Figure 2:**
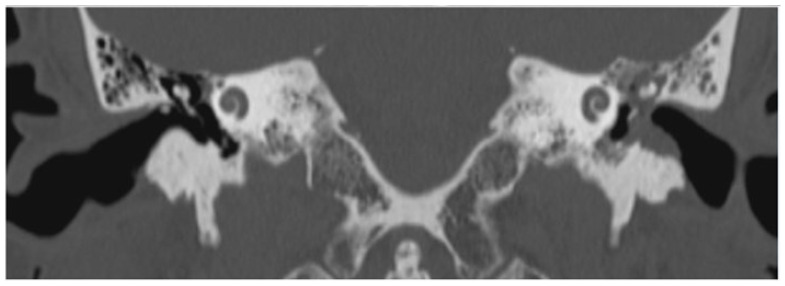
Patient 1, CT scan: evidence of soft tissue density located in the hypotympanic region of the middle ear cavity and in the floor of the left EAC with related bone erosion of the anteroinferior wall of the EAC.

High-resolution computed tomography scan of the temporal-bone showed a soft tissue density located in the hypotympanic region of the middle ear cavity and in the floor of the EAC, with related bone erosion of the anteroinferior wall of the left EAC. The lesion did not cause erosion or mass effect on the ossicular chain (Figure 2).

The injury, causing erosion of the anteroinferior wall of the EAC and extension to the adjacent temporo-mandibular joint (TMJ), without complications, was staged as IIIa, according to the classification of HN et al. [1] (corresponding to bone erosion—stage III reported by Naim et al. [2]).

The patient underwent a trans-canal endoscopic ear surgery (TEES) approach using a rigid endoscope with a 3 mm diameter and 0° viewing angle to explore the tympanic cavity and a canaloplasty to remove the lesion located on the EAC anteroinferior wall. During the exploratory tympanotomy, the ossicular chain and the tympanic cord were identified, and mucous secretions were aspirated. The tympanic cavity was free from cholesteatomatous disease. The tympanic perforation was reconstructed by myringotomy. The patient underwent endoscopic canaloplasty with reconstruction with fascia. The final histology of the removed lesion on the EAC inferior wall revealed EACC.

### 3.2. Patient 2

In March 2022, a 65-year-old male presented with a long history of right otorrhea and bilateral hearing loss, more severe in the right ear. He had already undergone antibiotic therapy without clinical benefit. The patient also reported a history of nasopharyngeal undifferentiated carcinoma diagnosed when he was 46 and treated with chemoradiotherapy with a total dose of 70.2 Gy in 39 fractions, with concomitant cisplatin.

The physical examination with otomicroscopy revealed both tympanic membranes intact, the right one slightly opaque. Furthermore, in the right ear, bone erosion of the inferior wall of the EAC was found.

The audiometric exam revealed a moderate degree of neurosensory hearing loss on the left (PTA equal to 55 dB HL) and severe degree on the right (PTA equal to 70 dB HL) with bilateral loss of frequencies above 2000 Hz.

**Figure 3:**
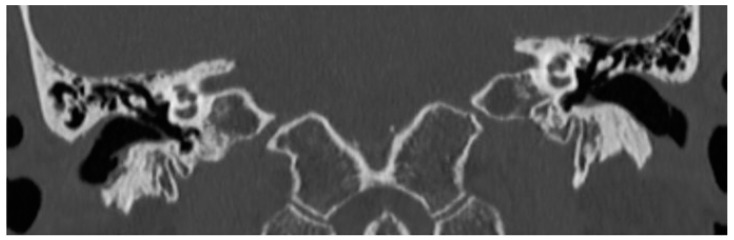
Patient 2, CT scan: evidence of a thin layer of soft and erosive tissue on the right inferior wall of the EAC.

The high-resolution computed tomography scan of the temporal-bone showed a thin layer of soft and erosive tissue on the right inferior wall of the EAC. The lesion did not involve the middle ear cavity (Figure 3).

**Figure 4:**
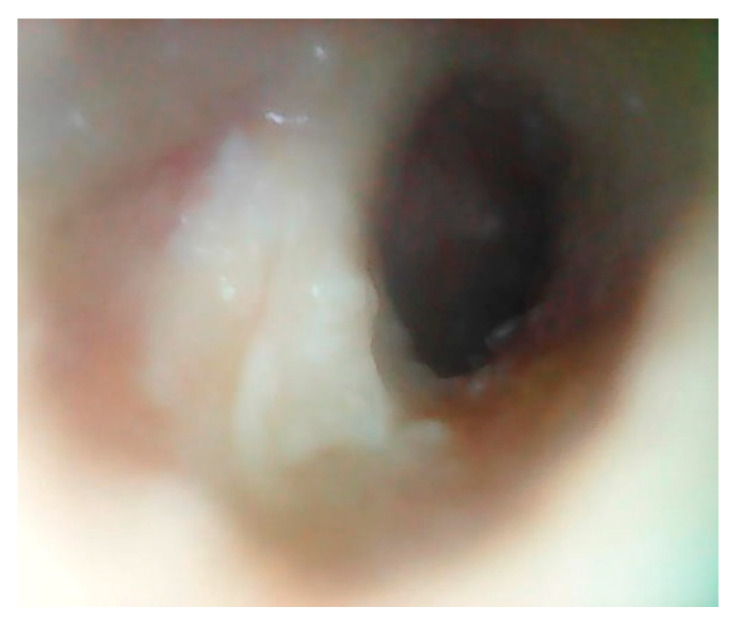
Preoperative image (bone erosion of the EAC inferior wall).

The lesion was staged as II according to the classification of HN et al. [1] (corresponding to periostitis—stage II reported by Naim et al. [2]), as shown in Figure 4.

**Figure 5:**
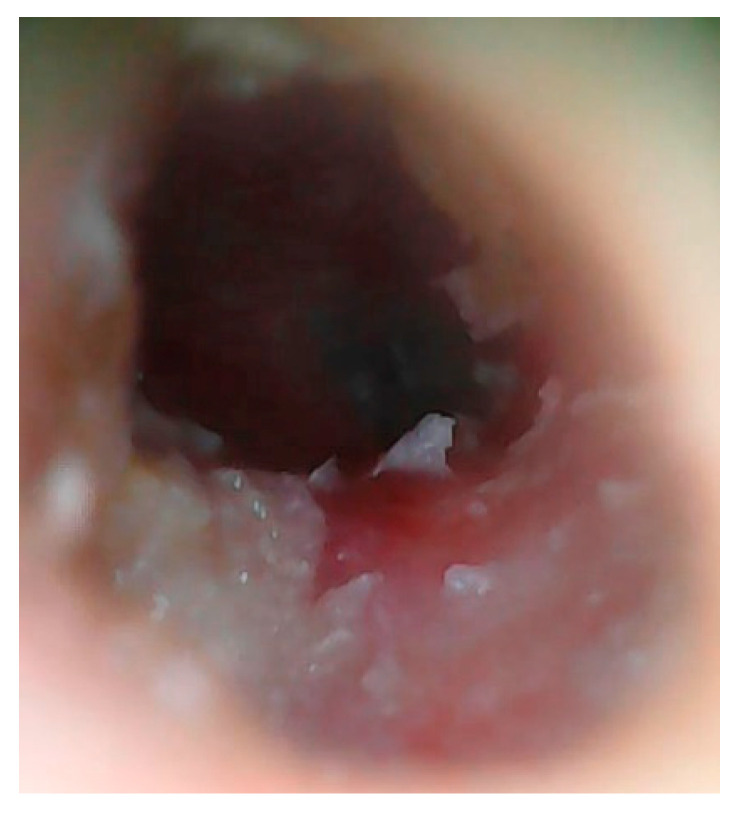
Post operative image 3 months after TEES (adequate re-epithelialization of the EAC.)

The patient underwent a TEES approach using a rigid endoscope with a 3 mm diameter and 0° viewing angle for the EAC lesion resection and consequent reconstruction with fascia. The final histology revealed EACC. The clinical evaluation 3 months after TEES highlighted adequate re-epithelialization of the EAC (Figure 5).

### 3.3. Patient 3

In February 2022, a 64-year-old male presented with a history of left ear otorrhea, unresponsive to antibiotic therapy after several months. The patient also reported a history of nasopharyngeal undifferentiated carcinoma diagnosed when he was 44 years and treated with RT with a dose of 66 Gy in 30 fractions.

The physical examination with otomicroscopy revealed a lesion of the anterior wall of the left EAC.

The audiometric exam revealed a moderate degree of neurosensory hearing loss on the right (PTA equal to 57 dB HL) and a severe degree on the left (PTA equal to 88 dB HL), with bilateral loss of frequencies above 4000 Hz. 

**Figure 6:**
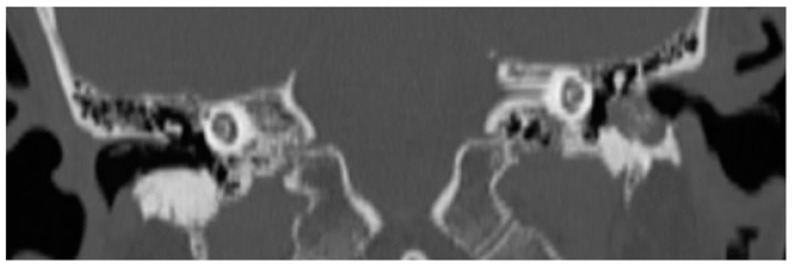
Patient 3, CT scan: evidence of soft and erosive tissue on the anterior wall of the left EAC.

The high-resolution computed tomography scan of the temporal-bone showed a soft and erosive tissue on the anterior wall of the left EAC. The lesion did not involve the middle ear cavity (Figure 6).

The lesion, causing erosion of the anterior wall of the EAC and extension to adjacent TMJ without complications, was staged as IIIa, according to the classification of HN et al. [1] (corresponding to bone erosion—stage III reported by Naim et al. [2]).

The patient underwent the removal of the erosive lesion and the peri-wound tissue through TEES, using a rigid endoscope with a 3 mm diameter and 0° viewing angle, after which a reconstruction with fascia was performed. 

The final histology revealed an EACC.

### 3.4. Systematic Review

**Figure 7:**
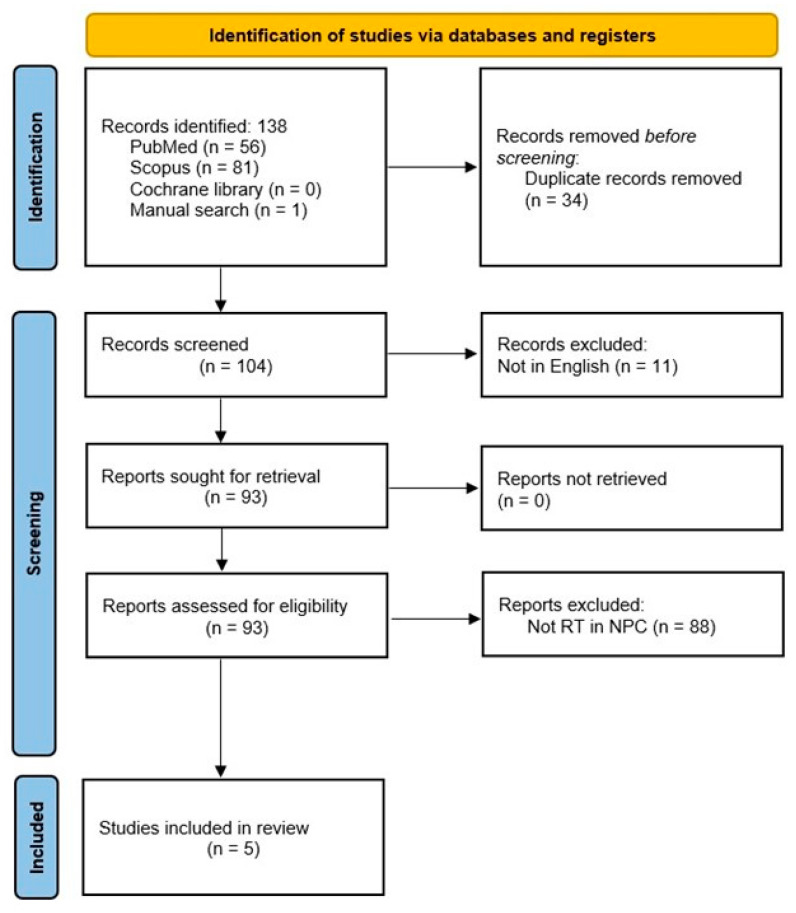
Search Strategy.

The search strategy was performed according to the PRISMA guidelines as shown in Figure 7. Overall, 138 papers were identified and, after duplicate removal (34 papers) and excluding papers not written in English, 93 papers were assessed for eligibility; finally only five papers were included and summarized in Table 1 together with the three cases coming from our institution [4,13,14,15,16].

### 3.5. Bias

All papers included were retrospective case reports of case series.

According to the Oxford Levels of Evidence [12], the case studies were level 4.

### 3.6. Patient Characteristics

Overall 23 patients were identified, including those in the present report.

There was a slight male prevalence and in most cases the mean age was in the fifth decade, but patients between 28 and 75 years old have been described. 

In most cases, there was the involvement of the anterior and the inferior part of the auditory external canal; in 26% of the cases, EACC was bilateral.

For the mean time of diagnosis after RT, the largest series was 6.5 years (with a range from 0.5 to 15.4 years). The total dose ranged from 42 Gy to 74 Gy and, with regard to the techniques used, either 2D or IMRT were reported.

## 4. Discussion

EACC represents a relatively unusual disease as compared to middle ear cholesteatoma. This pathology may be spontaneous (primary EACC, with unknown etiology), or consequent to other conditions (secondary EACC) [17].

The possible causes of secondary EACC described are chronic inflammation, stenosis, late complications of temporal bone trauma, ear surgery, and radiation therapy [18].

Our cohort consists solely of males with unilateral pathology involving predominantly the anterior and the inferior parts of the bony EAC, in line with the papers of Yu et al. [4], Sapmaz et al. [13] and Lin et al. [18].

The responsible factors for EACC development suggested in the literature were hypoxia and the expression of angiogenic factors [19].

After RT, a decrease in blood vessels associated with fibrosis increase within the irradiated skin area may occur [20].

Yu underlines that the EAC floor is characterized by a lower vascularization than the vascular strip of the EAC roof. Therefore, this may explain why the predominant invasion of RT-related EACC is in the inferior part of the EAC [4].

The effects of RT on the EAC have been a matter of great interest for several decades; in fact, the pathologic changes noted may be divided into two distinct categories: bony changes and soft tissue changes [21].

RT for nasopharyngeal cancer is by far the most relevant clinical example for secondary EACC development, because typically these patients are young, have good prognosis and receive high doses to large volumes [22].

Up to 13.8% of patients receiving RT for head and neck tumors are expected to develop external ear radiotherapy-induced morbidity [23].

The available evidence is too scarce to draw any definitive conclusion; however, apparently the initial stage of the NPC does not seem to influence the risk of developing secondary EACC [4].

However, it is reasonable to believe that the lower doses delivered by volumetric techniques, which allow the sparing of normal tissue as much as possible, should be associated with lower incidence of EACC compared to older techniques such as 2D [24].

In addition, it is desirable that modern techniques such as Intensity modulated radiotherapy (IMRT) for nasopharyngeal cancer should reduce post-irradiation external ear disorders’ late toxicity, possibly by identifying clinically validated dosimetric constraints [25].

The most commonly reported side effects associated with EACC after RT include otalgia, fullness and itching [26].

In our experience, the cardinal symptoms of RT-related EACCs were otorrhea, hearing loss and otalgia. Similar studies by Hn et al. [1], Dubach and Häusler [27] and Helibrun et al. [28] showed that otorrhea, otalgia, and hearing loss represent typical EACC onset symptoms. Otorrhea and otalgia are consequent to the local invasion of squamous tissue into the bony EAC [29].

Owen et al. describe that the average presentation latency of ear side effects due to RT is 5 years (range, 3–12 years) [26]. In the paper of Yu et al., the average latency is 6.5 years (range, 0.5–15.4 years) [4]. There are also reports with a presentation latency greater than 15 years [30].

In our series, the average presentation latency of EACC was 14 years after RT for nasopharyngeal carcinoma with a range between 9 years and 20 years.

We decided to stage the EACCs according to the HN [1] and Naim [2] classifications; however, in the literature there are other proposed systems specifically related to the radiological aspect of the EACC [31].

The surgical strategy used for these three patients was TEES, as its efficacy and security regarding the exposure and eradication of both primary and secondary EACCs is widely described [32,33].

In particular, for EACC limited to the EAC (up to Naim stage III), TEES represents the appropriate surgery, as tissue damage is minimized and consequently the median healing time decreases as compared to retro-auricular or endaural surgical approaches [34].

Moreover, an endoscopic approach to canaloplasty is described as safe and minimally invasive with significantly fewer postoperative complications as compared with microscopic procedures.

The 0° viewing angle allowed optimal visualization of the tympanic membrane in all three cases presented, in line with the larger series published in the literature [35].

An endoscopic approach allows an improved view of the end of the instruments, a minimally invasive approach and a decreased operative time [36].

## 5. Conclusions

Our systematic review of EACC after RT for nasopharyngeal cancer was limited to retrospective case series and case reports. Only 20 patients were identified in the literature. Therefore, the scantiness of data does not allow the drawing of any conclusive evidence. However, to our knowledge, this is the first systematic review to examine EACC incidence in patients receiving RT for nasopharyngeal cancer.

## Figures and Tables

**Table 1:** Main features of the studies included in the present systematic review.

Author	Year	Country	n. of Pts	Age	M/F	RT Dose	RT Techinque	Time from RT	Side	Location
Mammarella et al.	2023	Italy	3	62 (range 57 to 64)	3/0	66 to 70.2 Gy	2D	14 years (range 9 to 20 years)	2 left 1 right	a and i
Sapmaz et al. [13]	2016	Turkey	1	55	0/1	74 Gy	n.a.	4 years	Bilateral	a and i
Yu et al. [4]	2015	Taiwan	15	55 (range 32 to 75)	11/4	42 to 73.8 Gy	I.M.R.T. 2D	6.5 years (range 0.5 to 15.4 years)	7 left 5 right 3 bilateral	a and i
Zahara et al. [14]	2013	Indonesia	1	39	0/1	n.a.	n.a.	2 years	Bilateral	n.a.
Bennet et al. [15]	2007	U.S.A.	1	53	0/1	n.a.	n.a.	9 years	left	n.a.
Smouha et al. [16]	1995	U.S.A.	2	29 (range 28 to 30)	1/1	70 Gy	n.a.	n.a.	1 bilateral 1 left	n.a.

a: anterior; i: inferior; n.a.: not available; I.M.R.T.: Intensity Modulated Radiation Therapy; RT: Radiation Therapy.
